# Understanding participation in European cohort studies of preterm children: the views of parents, healthcare professionals and researchers

**DOI:** 10.1186/s12874-020-01206-5

**Published:** 2021-01-12

**Authors:** Sandra C. S. Marques, Julia Doetsch, Georgia Abate, Anne Brødsgaard, Grazia Colombo, Marina Cuttini, Pernille Pedersen, Henrique Barros, on behalf of RECAP Preterm-WP6 QS Work Group, Sandra C. S. Marques, Julia Doetsch, Raquel Teixeira, Georgia Abate, Grazia Colombo, Marina Cuttini, Anne Brødsgaard, Elizabeth S. Draper, Sylvia van der Pal, Ilona Wildeman, Pernille Pedersen, Kari Anne I. Evensen, Ann-Mari Brubakk, Marit S. Indredavik, Eero Kajantie, Eeva Virtanen, Jo Lebeer, Vicky Hennissen, Iemke Sarrechia, Henrique Barros

**Affiliations:** 1 EPIUnit, Instituto de Saúde Pública da Universidade do Porto, Rua das Taipas 135, 4050-091 Porto, Portugal; 2 Centro em Rede de Investigação em Antropologia, Instituto Universitário de Lisboa, Lisboa, Portugal; 3 Clinical Care and Management Innovation Research Area, Bambino Gesù Children’s Hospital, IRCCS, Rome, Italy; 4 Department of Paediatrics and Adolescent Medicine, Copenhagen University Hospital Amager Hvidovre, Hvidovre, Denmark; 5 Department of Public Health, HEALTH, Aarhus University, Aarhus, Denmark

**Keywords:** European cohorts, Longitudinal, Preterm children, Participation, Retention, Multi-situated qualitative study, Collaborative visual methods

## Abstract

**Background:**

Retention of participants in cohort studies is a major challenge. A better understanding of all elements involved in participation and attrition phenomena in particular settings is needed to develop effective retention strategies. The study aimed to achieve an in-depth understanding of participant retention in longitudinal cohorts focusing on participants’ and researcher’s perspectives, across three diverse socio-geographic and cultural settings.

**Methods:**

This study used a triangulation of multi-situated methods to collect data on cohort studies of children born with less than 32 weeks of gestation in Denmark, Italy and Portugal. It included focus groups and individual semi-driven interviewing with involved key actors (i.e. parents, staff, healthcare professionals, researchers) and a collaborative visual methodology. A purposive sample of 48 key actors (*n* = 13 in Denmark; *n* = 13 in Italy; *n* = 22 in Portugal) was collected. A triangulation of phenomenological thematic analysis with discourse analysis was applied. Cross-contextual and context-specific situational elements involved in participation and attrition phenomena in these child cohorts were identified at various levels and stages.

**Results:**

Main findings included: situational challenges affecting potential and range of possibilities for implementation strategies (geopolitical environment, societal changes, research funding models); situational elements related to particular strategies acting as deterrents (postal questionnaires) and facilitators (multiple flexible strategies, reminders, regular interaction); main motivations to enrol and participate (altruism/solidarity and gratitude/sense of duty to reciprocate); main motivational deterrents to participate to follow-up waves (lack of bonding, insufficient feedback); entanglement of clinical and research follow-up as facilitator and deterrent.

**Conclusions:**

The multi-situated approach used, addressing the interplay of the lived experience of individuals, was of most value to understand participation variability under different implemented strategies in-context. Cross-contextual and context-specific situational elements that have been influential factors towards participation and attrition in the cohorts were identified.

**Supplementary Information:**

The online version contains supplementary material available at 10.1186/s12874-020-01206-5.

## Background

Population-based cohort studies are a powerful research design to understand human life-course development and causal mechanisms [1, 2]. Over the years, these studies have importantly contributed to our understanding of disease trends, predisposing and protective influences, and susceptibility during life course transitions. The increased use of networks of multiple, long-term cohort studies has also the potential to capture the value and differential effect of policy and program interventions that operate within and outside the health sector on the health quality and health equity of populations [3].

Cohorts are complex structures that require continued involvement of both cohort participants, and researchers, ongoing funding and supporting infrastructure to ensure continuous attention to timeliness, attrition and quality of collected information. Those requirements are indispensable to meet high scientific standards and to allow the appropriate translation of findings into clinical practice and policy actions. The success depends, not only on the initial adequate enrolment of participants, but also on their sustained response to subsequent data collection waves over time. Retention of participants is a major concern and a well-known challenge. Approaches to this issue necessarily vary according to international specificities in research regulations and contextual differences.

Available evidence from the past decades suggests that researchers should consider the use of multiple strategies to enhance retention. Financial incentives have been associated with an increase in retention proportional to the incentive value. Relevant increases in retention were also associated with the offer of alternative locations and modes of data collection, repeat postal questionnaires, reminder letters and telephone calls [4]. Targeted strategies, such as incentives to non-responders from previous waves of the study, were also reported as a cost-effective approach for retention. Moreover, regular contact between researchers and participants enhances bonding and enduring identification with the study [5].

Notwithstanding, it has been shown that participation in cohort studies has been decreasing over the past three decades [6]. Findings are yet constrained by small number, geographical concentration, scarce details and inconsistent description of published studies reporting implemented retention strategies, which restricts inferential leaps or generalization to other populations and settings. Subsequently usefulness of proposed retention strategies may vary [7]. Recently, an extensive review has found that follow-up incentives such as cash, repeat questionnaires and reminders, the most commonly used strategies, were associated with poorer retention. The merely addition of more cohort retention strategies also seemed not to result in higher retention rates [8]. Further primary research is needed, therefore, to expand the population assessed, diversity of studies and settings to better understand variability.

While knowledge on the perspective of study participants and their motivations for taking part in cohort studies for different settings and populations are essential to inform researchers on recruitment and retention methods, the available information is scarce [9]. Longitudinal studies with high retention rates commonly used personalized approaches and tailored retention strategies specifically to their cohorts [10]. It is also known that behavioural decision-making is more complex, fluid and situational than what may be assessed through quantitative cost–benefit analysis of probabilities as it is dependent on individuals’ personal traits, situational emotional responses and lived experience [11].

Hence, knowledge on perceptions and experiences of diversified participants in various contexts and study approaches with attention to the interplay of the lived experience in both researcher and researched cohort stances are needed to better understand the situational elements that influence retention [12]. The study aimed to achieve an in-depth understanding of participant retention in longitudinal cohorts by focusing on the interplay of both participants’ and researchers’ perspectives, motives and lived experiences across three diverse socio-geographic and cultural European settings.

## Methods

### Project participants

The study was developed under the” Research on European Children and Adults born Preterm” project (RECAP Preterm), which joined 20 population-based cohorts from 13 European countries, assembling data of very preterm and/or very low birth weight (VPT: < 32 weeks of gestation /VLBW: < 1500 g) individuals followed since birth.

In this paper we evaluated three subordinate cohorts from the studies “Effective Perinatal Intensive Care in Europe” (EPICE) and “Screening to Improve Health in Very Preterm Infants in Europe” (SHIPS) of RECAP Preterm consortium: i) EPICE/SHIPS-DK from Denmark (DK); ii) EPICE/SHIPS-IT from Italy (IT); and iii) EPICE/SHIPS-PT from Portugal (PT), which include children born with less than 32 weeks of gestation in 2011–12, recruited and followed-up under common pre-established protocols. VPT babies were recruited at the neonatal intensive care units (NICUs) and followed up until discharge. NICU survivors were followed-up at 2 years of age via postal questionnaires to obtain information based on parental assessments [13]. The SHIPS project built on the EPICE project and assessed the cohorts at 5 years of age, using: 1) postal questionnaires to obtain information on parental assessments; 2) in-depth semi-structured individual interviews to a sub-sample of 10–15 carers of children; 3) a neurodevelopmental assessment of the sub-set of children born < 28 weeks GA (Table 2).

### Study design

This study was based on a established Study Protocol already published [14] (Supplementary file 1). Purposive non-probability sampling was used to achieve a socio-geographic heterogeneous sample of parents of cohort children, including parent organisation representatives (Ps), healthcare and research professionals involved with VPT/VLBW cohorts (PRFs). Participants were contacted and enrolled with the collaboration of each partnering cohorts’ research team.

This study applied qualitative research following a phenomenological analysis with an idiographic (representational) focus. Thus, it aims to provide insights into how a given person, in a specific context, makes sense of a given phenomenon. It is focused on the meaning of behaviour, narrative and the “lived personal experience” [15].

A multi-situated method was used to collect data. It comprises both the concept of multi-sites (or multi-locations) and of situated knowledge [16]. “Situated knowledges” imply the significance of the material, social and political conditions that enable multiple, partial, diverse knowledges at a given moment as well as the responsibility to consider them just as valuable [17]. The framework as described in detail in the Study Protocol [14] resorts to a triangulation which includes several qualitative data collection methods: i) focus groups, ii) individual semi-driven interviews, iii) and a collaborative reflexive visual methodology (VideoStories). VideoStories is a collaborative methodology using participant-generated videos and video debriefing interviews. It derives from photo/videovoice process grounded in phenomenology and hermeneutics [18, 19]. Individuals were expected to reflect more in-depth and communicate their “lived personal experience” and acquired knowledge as research participants through and alternative way of expression. The inclusion of this method is particularly advantageous to potentiate both barrier-reduction and inclusiveness of hard-to-reach participants and to promote a more participatory relationship. Participants benefit by having the opportunity to represent themselves in the research process and in its findings while researchers benefit by their engagement, potentiating retention and identification to the cohort studies [12].

### Data collection

Following the epistemological principle of valuing multi-situated knowledge, research partners chose and combined from proposed methods those most pertinent to their particular contexts and targeted participants. Multi-site sub-samples and their sizes varied therefore within the range pre-established by the shared protocol. The total sample contributing to these findings comprised 48 participants (*n* = 13 in Denmark; *n* = 13 in Italy; *n* = 22 in Portugal): 26 parents of cohort participating children aged 6–8, including individuals who had failed to respond to previous waves of the studies, and 22 involved professionals (Table 1). The majority (83,3%) of the sample was female: PRFs (*n* = 20/22) and Ps (*n* = 20/26); ages ranged from 25 to 65 years (PRFs: 25-65 yrs. and Ps: 25-50 yrs).

**Table 1:** Implementation of context specific methods

Country	Cohorts	Method	Number of participants
Denmark	EPICE/SHIPS-DK	II with Healthcare and research professionals	*n* = 2
		FG with Healthcare and research professionals	*n* = 4
		II via telephone with Parents^a^	*n* = 7
Italy	EPICE/SHIPS-IT	FG with Healthcare and research professionals	*n* = 6
		Two FG with Parents	*n* = 5
		II via telephone with Parents^a^	*n* = 2
Portugal	EPICE/SHIPS-PT	II with Healthcare and research professionals	*n* = 3
		FG with Healthcare and research professionals	*n* = 7
		Three FG with Parents (including Parents^a^)	*n* = 11
		13 VS with Parents	*n* = 6
		Video debriefing interviews	*n* = 5
Total			*n* = 48

Legend: The Table described the implementation of the context specific methods in each country and their cohort accordingly

Note on abbreviations:

*II* Individual semi-structured interview, *FG* Focus Group discussion, *VS* Video Stories

^a^parents who failed to respond in previous follow-up waves

We have selected an exploratory approach to potentiate the free emergence of new concepts in the discussions. The moderator/interviewer was, therefore, as non-directive as possible [20]. A commonly defined guide of 6–8 key-issues to approach was used only as discussion triggers and if not spontaneously approached by participants. In all sites, it was firstly conducted one focus group discussion with professionals which was also used to explore and adjust for specific sub-themes to probe in further group and/or individual semi-driven interviewing with professionals and with parents (Supplementary material 1). Additional focus groups, individual interviews and/or VideoStories were conducted until saturation was achieved. VideoStories participants were given a common task of generating 3–4 short videos during a similar period of time after which a video debriefing interview was conducted lasting 90 min. on average. Focus groups lasted on average 2 h with at least two researchers present and individual interviews had an average length of 30 min.

Data were collected between April 2018 and March 2019 in the country’s official language. Written explicit consent was retrieved from all participants. Data were audio recorded, transcribed and translated into English.

### Data analysis

A triangulation of phenomenological thematic analysis with discourse analysis was used to analyse the data. Visual and verbal depictions were both treated as narratives. The first principle is to use an emergent strategy, to allow the method to follow the nature of the data itself which may emerge or change in the course of analysis. Therefore, sub-sets of data were sorted and categorized by hand by a team of two researchers led by a social scientist experienced in this kind of analysis for multimodal data. Thematic analysis was used to determine if any patterns or representational axes emerged from recurrent themes and repetitions (discursive formations) as well as relevant deviances. Emerged themes and representational axes at several stages were discussed, refined and further verified with multi-site partnering research teams. Two types of themes developed: i) collective themes, occurring across a large number of participants in different settings; and ii) context-specific themes, unique to certain individuals or settings. Additional information to triangulate our results was gathered from: cohorts’ management teams via internal survey, meetings and email, and cohort studies’ publications. A final interpretative analysis of relevant elements involved in the phenomena of participation and attrition both in particular and across cohorts was undertaken by the generic application of the mode of contents contingency.

## Results

Results display major elements involved in the phenomena of participation and attrition and interplay of standpoints/perspectives found. Following the epistemological principle of conveying situated knowledges, they are situated (contextualized/interrelated) within relevant surrounding conditions that have enabled their construction.

### EPICE/SHIPS: one European study, three different approaches

Losses due to failure to locate, contact or to respond due to burdensome or unsuitable follow-up procedures emerged as major concern for professionals in all settings (*n* = 22/22). The three cohort management teams variously implemented multiple strategies to interact with participants and locally apply shared EPICE and SHIPS protocols, having modified and adapted strategic procedures over the cohort’s follow-up to maximize retention. In PT, the frequency of strategic monitoring was increased to annual. PT also extended the neurodevelopmental assessment at 5 years to the whole VPT cohort, combining, at same time and location, the administration of the parental questionnaire. DK, though performing both pre-established follow-up protocols, did not participate in the 5-yrs assessment through face-to-face interviews (Table 2).

**Table 2:** Cohorts’ recruitment and EPICE/SHIPS study waves in Portugal, Italy and Denmark: strategies of implementation and participation

PT - Portuguese Cohort			IT - Italian Cohort		DK - Danish Cohort			
Regions: Northern; Lisbon and Tagus Valley			Regions: Emilia Romagna; Marche; Lazio		Regions: Funen; Zealand; Lolland; Falster			
EPICE study								
Cohort recruitment Birth/baseline 2011/12	F-to-f by neonatologists of the units where the babies were born/being cared, at the hospital unit. 1. data abstracted from medical records	Participation: 544^a^/607 d.a. (90%) (*n* = 879 live & still births)	F-to-f by neonatologists of the units where the babies were born/being cared, at the hospital unit. 1. data abstracted from medical records	Participation: 975 /975 d.a. (100%) (*n* = 1326 live &	F-to-f by neonatologists of the units where the babies were born/being cared, at the hospital unit. 1. data abstracted from medical records still births)	Participation: 286/286 d.a. (100%) (*n* = 441 live & still births)		
F-up at 1 yr ca 2012/13	1. parental questionnaire by phone.	Participation: (84%)	(did not take place)		(did not take place)			
F-up at 2 yrs. ca 2013/14	1. parental questionnaire sent out by post (and to be returned by post).	Participation: (75%)	1. parental structured questionnaire sent out by post and/or email (and to be returned by post or email) or admin. by phone, if necessary.		Participation: (75%)	1. parental structured questionnaire sent out by post (and to be returned by post).		Participation: (63%)
F-up at 3 yrs. 2014/15	1. parental questionnaire by phone. 2. parental questionnaire (3d’ food diary + CBCL) sent out by post (and to be returned by post).	Participation: (87%)	(did not take place)			(did not take place)		
F-up at 4 yrs. 2015/2016	1. parental structured questionnaire by phone.	Participation: (83%)	(did not take place)			(did not take place)		
SHIPS study								
F-up at 5 yrs. 2016/17	1. structured questionnaire by parents (on site, while children were being tested).	Participation: 435/533 (82%)	1. parental structured questionnaire sent out by post and/or email (and to be returned by post or email) or admin. by phone, if necessary.	Participation: 692/975 (71%)	1. parental structured questionnaire sent out by post (and to be returned by post).		Participation: 152/286 (53%)	
	2. neurodevelopmental assessment by a team of psychologists and nurses at alternative locations (results sent to parents or to referred paediatrician according to parents’ preference).	Participation: (82%) (EPT *n* = 113)	2. neurodevelopmental assessment of the sub-sample extremely preterm (EPT) by a team of psychologists at alternative locations (results handed over to parents).	Participation: 135/223 EPT (60.5%)	2. neurodevelopmental assessment ofthe sub-sample extremely preterm (EPT) by a team of physiotherapists and psychologists at alternative locations (results handed over to parents).		Participation: 42/52 EPT (81%)	
	3. F-to-f in-depth semi-structured interviews to a sample of 10–15 parents of the sub-set EPT that completed the questionnaire, at alternative locations.	Participation: *n* = 12	3. F-to-f in-depth semi-structured interviews to a sample of 10–15 parents of the sub-set EPT that completed the questionnaire, at alternative locations.	Participation: *n* = 14	(did not take place)			
Source:	EPICE/SHIPS-PT cohort research team, Dec 2019.		EPICE/SHIPS-IT cohort research team, Nov 2019.		EPICE/SHIPS-DK cohort research team, Sept 2019.			

Legend: This Table gives an overview on the cohorts’ recruitment and their strategies of implementation and participation of the EPICE/SHIPS study waves in Portugal, Italy and Denmark

*d.a*. discharged alive from hospital

^a^52 parents were not invited to participate in the cohort; 11 refused follow-up

Available taxonomic systems for categorizing retention strategies vary, reflecting the inadequacy of classifying those serving multiple purposes and uncovering the widespread inconsistencies in results. Range and divergence of procedures specifically aimed to maximize retention by the three cohorts were here grouped into four domains following Teague et al. (2018) [8].

(i) “Barrier-reduction strategies” included: assistance with postage costs (PT; DK; IT); flexibility for phone contact and scheduling at evening time (DK) and weekend (PT; IT); offer of alternative methods for data collection, e.g. administration of questionnaires by phone (PT; IT), and their return in digitalized form (IT). At the 5-yrs-follow-up, a neurodevelopmental child assessment and face-to-face interviews took place. Participants were offered assessments at home or close by, and cater/refreshments for those who travelled (PT;IT;DK). Assistance with transport (cost refunding) and lodging was also offered to families living outside the city, or on demand (IT).
(ii) “Bond-building strategies” included a common website of the European projects in English language displaying related news, publications, and at the 5-yrs-follow-up also individual feedback on the neurodevelopmental assessment (individual report). An age-appropriate book as a gift to the children was either mailed or offered at the end of the neurodevelopmental assessment (IT). An annual birthday postcard to the children, a book on EPICE-PT study results sent to parents in 2015 and a newsletter (though not regular) in Portuguese, sporadic emails on media appearances related with the cohort and two gathering events of participants in Porto and Lisbon were implemented (PT).
(iii) “Reminders” and “other strategic incentives to improve participation within each study wave” included phone calls, letters, emails and/or phone text messages reminding to respond to the wave events assessed by questionnaires (PT;IT;DK). Financial incentives, e.g. cash, vouchers or rewards to complete assessments within data collection waves, were not used by any of the cohorts.
(iv) “Tracing and contact strategies”, mainly included: postal mail, email, and/or phone call, while resorting to the cohorts’ database and by trying to keep contact details updated for each participant. Resorting other database locators as per the possibilities allowed by national regulations and available systems was attempted when facing difficulties (PT;IT;DK). The PT cohort also combined the procedure of updating multiple modes of contact, yearly, through the aforesaid annual monitoring strategy (Table 3).

**Table 3:** Retention strategies applied by the three cohorts throughout studies’ waves

EPICE/SHIPS Studies			
Cohort recruitment Birth/baseline: 2011/12; Most recent follow-up in all cohorts at 5 yrs.: 2016/17			
Retention Strategies	PT	IT	DK
	Cohort	Cohort	Cohort
(i) “Barrier-reduction strategies”			
• assistance with postage costs	✓	✓	✓
• flexibility for phone contact and scheduling	✓	✓	✓
• offer of alternative methods for data collection	✓	✓	✓
• offer of home or closer to home assessments	✓	✓	✓
• offer of catering / refreshments	✓	✓	✓
• assistance with transport and lodging costs (occasionally)		✓	
(ii) “Bond-building strategies”			
• common dedicated European website (English language)	✓	✓	✓
• related news and published study documents in the website	✓	✓	✓
• sharing individual feedback on study results (of the neurodevelopmental assessment at 5 yrs)	✓		✓
• book on EPICE study results sent to parents (country’s language)	✓		
• age-appropriate book offered/sent to children (at the 5 yrs. study wave)		✓	
• annual birthday postcard sent to children	✓		
• newsletter (country’s language)	✓		
• emails on media appearances related with the cohort (sporadic)	✓		
• gathering events with researchers / other participants	✓		
(iii) “Reminders/other extra incentives to participation within each study wave”			
• reminder phone calls	✓	✓	✓
• reminder letters	✓		✓
• reminder emails and/or sms	✓	✓	
• cash incentives / voucher incentives / specific rewards for assessment completion at the wave event			
(iv) “Tracing and contact strategies”			
• tracing via cohort database by postal mail and alternative contacts: email and/or phone call	✓	✓	✓
• alternative tracing via healthcare/institutional tracking database system	✓	✓	✓
• tracing via update your contact details (annual monitoring contact)	✓		
• tracing via public records /network focal points	✓	✓	✓

Legend: This table demonstrates the retention strategies applied by the three cohorts throughout studies’ waves

#### Situational challenges to EPICE/SHIPS cohort teams

Professionals’ and parents’ perspectives converged in all sites on desirable traits of strategies to contact and interact with cohort participants (PT;IT;DK). As reported: a) flexibility to reconcile agendas by offering alternative methods and contact timing; b) availability to bring the study closer to participants by providing appropriate location and language mediators, and assistance with incurred costs; c) bonding enhancement, through sharing of research results with participants, and promoting communication bridges.

However, situational challenges affecting the potential of implemented strategies were reported by professionals in all cohorts even though research teams implemented somehow context-sensitive approaches (PT;IT;DK).

As described, the fast rhythm of societal changes regarding communication systems since the cohort’s recruitment in 2011–12 has hampered the efficiency of available tracing systems to reconnect to cohort participants after one loss of contact. Two most influential deterrents were stressed by all Professionals: a) increased informatization of databases and work processes with replacement of systems at times asynchronous and discordant; b) increased constrained access to personal data and possibility of record linkage due to legislations and regulations (PT;IT;DK). The impacts of the progressive dismissal of home phone landlines and the increasing reliance on mobile/electronic contacts detached from physical addresses within the last decade was also emphasized across IT and PT contexts.

*(…) families move often so it is a problem to find the address, you need to contact the registry office; the cell phone numbers change frequently and the landline no longer exist; very often the families change city or country especially the foreigners, thus to recover their information can be very complicated. To solve this problem, we can access the registry verification, through the municipal registration, or the regional database, making the process very slow (…) Even the email addresses can be an obstacle* (PRF5-IT).

*(…) if we have had a common [health database] system throughout the country, it had been easier (…)To open the journals of patients is not allowed without their consent now. (…) to find out what language the family speaks or… you can't just look from the name and address. So in this way it is a challenge to send correct questionnaires in the right language to the families* (PRF4-DK).

These children were recruited at NICUs, which are limited and centralized, and therefore may be located at long distances from the participants’ place of residence. Professionals reported that many participants became difficult to trace after discharge or the end of clinical follow-up at that same hospital (PT; IT; DK). Professionals in all settings shared common concerns that people from minority and vulnerable groups were at higher risk of loss to follow-up. Even when retraced, due to constraints in human and financial resources, it was difficult to provide context-sensitive methods, e.g. supporting long-distance travelling, involving interpreters, widening timing and providing alternative locations. Though stressed in all sites, the issue was specifically emphasized in the IT cohort, which is the largest.

Adding to these contextual barriers, it was also exposed that the study information provided to participants at recruitment and follow-up waves usually did not anticipate long-term future interactions, as these depend on prospective funding. Consent to participate in research must be restricted to a study protocol framed according to the short-term funding project. The dependency on impermanent funding further limited the possibilities to sustain regular contact in-between study waves and to meet parents’ expectations on promoting more bond-building strategies.

### Elements involved in the initial decision to enrol in VPT/VLBW birth cohort studies

#### Motivations

##### Altruism/solidarity and gratitude/sense of duty to reciprocate

The strategy of newborns’ enrolment at NICUs before discharge was very effective as reflected in the high level of recruitment achieved: 90–100% of all individuals born VPT discharged alive were enrolled in the EPICE study (DK cohort *n* = 286/286; IT cohort *n* = 975/975; PT cohort *n* = 544/607).

Two major concurrent representational axes emerged as main underlying motivating factors for high positive response from parents for enrolling (PT;IT;DK): a) “altruism/solidarity” and b) “gratitude/sense of duty to reciprocate”. “Altruism/Solidarity” related to the positive feeling of contributing to improve medical knowledge and health care practices to benefit preterm infants and families in the future. “Gratitude/Sense of duty to reciprocate” directed to healthcare professionals caring for their newborns in emotional challenging circumstances or redirected to other social counterparts that may benefit from that act.

*Mine is a choice of gratitude, to give my contribution to the research that helps preterm children born after mine, because I have benefited from it and therefore, I want to give something back* (P4-IT, mother).

Those two main underlying motivations were communicated even by parents who subsequently did not respond to any of the follow-up study waves.

##### Situational vulnerability of becoming parent of a VPT/VLBW child

In the NICU, the two overlapping roles as recruiter and as neonatologist taking care of these VPT newborns motivated parents to trust and consent to their enrolment. Both inquired parents and neonatologists involved acknowledged that the situational vulnerability of parents during those distressing circumstances represented an additional influential element in their decision-making.

*We recruited in the first days of life. (…) Saying that they are special babies and we can only improve our practices if we know what happens to these babies. And I think that at this point, parents listen to everything, they absorb everything, but at the same time, the emotional situation is so strong that they do not remember what they answered (…) when we talk later, they say: “Yes, I have an idea that you talked with me”* (PRF3-PT).

The lived experience of situational vulnerability at the time of enrolment emerged across parents’ accounts in all three settings, denounced by the recurrent expression of feelings of “fear”, “suffering”, “shock”, “trauma”, “despair”, “overwhelm” and “trying to cope”. These descriptions were associated with statements of vaguely or not remembering enrolment or not having retained information about its prospective trait. They were grateful for the provided medical care and just trusted in the medical/scientific community when asked to give back.

*(…) Coming from the doctor, I said yes. I did not ask for the mother’s consent, I said yes. At that time, I did not even think on worrying a mother about answering questionnaires, filling out reports, (…) [On what would be the study, its goals?] Zero! In that initial phase: zero! (…) It was a little like the other parents have said. It was very difficult to manage this situation. My son was born in a hospital emergency room and, as you may understand, I was extremely…* (emotion contained) *I panicked, I was angry too. (…) thanks to them my son survived.* (P11-PT, father).

Elements Involved in the Decision to Participate in Subsequent Follow-Up Waves of the Studies.

#### Motivations

##### Same leading motivations to enrol and to continue participating

The analysis of parents’ narratives showed a continuity in main leading motivations to enrol and to continue accepting the invitations for follow-up waves. “Altruism/Solidarity” and “gratitude/sense of duty to reciprocate” persisted as the main concurrent reasons provided to continue participating (PT; IT; DK). In Portugal, all parents (*n* = 12) also reinforced that if it had not been for the focus on providing data for the benefit of other parents and other children, they would have dropped out.

##### Entanglement of clinical follow-up and research follow-up

Participation in these cohort studies was found inextricably linked to parents’ lived experiences of having a VPT birth and of the healthcare and support provided to them and their children. As the initial enrolment was conducted at the hospital unit, clinical health monitoring and research follow-up experiences have been perceived as intertwined by all parents, even by those who expressed awareness of their independence (PT;IT;DK).

*We understood the difference after some time, and if we had not asked, we would have remained in doubt. (…) We found out later that the clinical follow-up is something different, (…), it is good for the child, but it is crazy that it does not serve also for research on these issues (…) our son did a lot of experimental treatments with the idea that any data collected that could help other children would be a good thing* (P5-IT, father).

Interestingly, this entanglement that had facilitated enrolment, became a deterrent for later follow-up uptake. All parents (*n* = 26/26) expressed difficulties in trying to cope with mandatory intense clinical appointments, therapies and treatments over the years. Persistent feelings of fear and being overwhelmed concurred in their narratives with the complaint of lack of healthcare provision of adequate psychological support for mothers (and families), particularly in the first two postnatal years (PT;IT;DK). Those mothers that failed to respond to follow-up waves (*n* = 7 DK; *n* = 2 IT), added descriptions of lived experiences of being mother of twins or more children, of severe child impairments, and single parenthood.

*(…) maybe the staff could had attached me to a psychologist or something. (…) I had a really hard time and I also had a really hard time when we got home and were still very sad. (…) if you are home and the boys are almost 1 year and I still could not talk about it without crying, so then it has been completely wrong inside I think* (P1-DK, mother of twins).

When reasoning about the motives for their decision, mostly referred to the importance of participating for the benefit of others. While one claimed not having received the invitation to that wave event, most stated not even remembering not having responded. According to them, researchers should have insisted (other time, other way) in obtaining their positive response (*n* = 7/9). Failure to participate was explained by “no surplus of energy” or “negligence” due to their persistent distressful, demanding lived experience of motherhood (*n* = 8/9).

##### Expectation of direct benefit for the child

Reinforcing the relevance of this perceived entanglement, a particular deviance was found amongst a few parents’ accounts in the Italian context. Three parents who clearly stated during the focus group discussion of not being aware of the independence of the cohort studies from the clinical follow-up of their children, pointed the expectation of direct benefit for the child as another main underlying motivation to have participated up to that moment. Notably, all of these parents also voiced their frustration/distrust in the healthcare system.

#### Motivational deterrents

Two major representational axes on demotivating factors for participation were abstracted from parents’ accounts.

##### Lack of bonding and of identification with the EPICE/SHIPS cohort studies

All parents revealed a lack of bonding and of identification with the cohort, although less evident in PT, where most intensive and extensive varied interaction with families was implemented. Parents’ main suggestions to improve bonding and identification with the cohort studies: 1) increasing cohort and follow-up visibility through media advertisement and amongst healthcare professionals; 2) regularly updated website on cohorts’ research findings and prematurity in each country’s’ language; and 3) regular communication via email or newsletter, and further consistency in interaction (DK;IT;PT). Other suggestions were context-specific to particular cohorts.

Several mothers in DK (*n* = 4/7) suggested that the research team should make use of obtained knowledge to support parents by sharing some tools on how to help/to handle VPT/VLBW in kindergarten and school. These mothers expressed their frustration on lack of professional support to raise awareness and understanding amongst teachers, educators, and the “commune” on why their children are so “stubborn”, “sensitive”, “explosive”, and “lack focus and attention”.

Almost all parents in IT (*n* = 6/7) suggested that follow-up should include clinical assessments of the children. All parents suggested to either synchronize it with clinical appointments or offer priority to access one as an incentive to enhance participants’ engagement to the cohort. The cross-contextual discontent with lack of adequate support for parents was stronger and multifocal in the IT cohort.

*(…) maybe it would be useful to offer (…) a preferential way if you need a [clinical speciality] visit, to gain time. The waiting lists are monstrous, so maybe it would be useful, since these children need a little more care* (P7-IT, mother).

Parents in PT singled out face-to-face, i.e. “closeness” to become familiar with the “faces behind the study” as the main facilitators to promote bonding and identification with the cohort (*n* = 6/12). Previous regular face-to-face interactions with researchers and other participants were declared as insufficient for an enduring engagement.

##### Insufficient information on the study and study findings

As also acknowledged by professionals, most parents confessed either not having retained any information on the enrolment or having forgotten about the study’s prospective trait. Insufficient information on recruitment and inadequacy in volume and frequency of further shared information were singled out by all parents as major demotivating factors for participation and reasons to feel disengaged (DK;IT;PT). Manifested preferences on expected regularly increased information sharing ranged from real testimonies of other parents, statistical information on the cohort, short conclusions of results between countries, to other relevant information such as policies, legislation, and “tools” to help parents.

*[Feedback matter to say yes in the future] because, then, I would feel a motivation, if I could see the outcome for what I have contributed. So, if I didn't hear anything, then I would feel that it wasn’t used for anything. Then I don’t know whether anything comes out of it or if it has any significance* (P5-DK, mother).

### Situational elements related to particular strategic procedures

Participants’ accounts revealed a variety of elements involved in the weighing process of decision-making to participate to specific wave events. As also perceived by professionals, all parents agreed that the use of multiple and flexible/tailored strategies to contact and interact with them favours participation (DK;IT;PT). As previously described, range and diversity of procedures and strategies aiming to maximize retention differed across cohorts. The PT cohort promoted most intensive and extensive varied interaction with participants over time and reported highest participation in the last assessment of the whole cohort (PT:82%; IT:71%; DK:53%).

The strategy of sending the annual birthday postcard particular to the PT cohort, was spontaneously introduced in the discussions both by professionals and parents as the most successfully implemented. As acknowledged by both, it had a suitable regularity to serve a number of cohort management purposes: participation reward; bonding enhancer for both parents and children; regular reminder of the study continuity; and, keeping regular updated postal contact. Most parents expressed that their children perceived it as an initiative directed to them – a bonding gift.

*[The postcards] are all there on the fridge. She loves it. It always comes after her birthday, but [she] loves the postcards* (P6-PT, mother).

Considering participation proportions in common follow-ups, parental structured questionnaires sent out and to be returned by post were associated with poorer response across the cohorts. In DK, where no alternative was offered, lowest participation was observed (Table 2). DK mothers that failed to respond to follow-ups indicated that this data collection method was the closing factor for their decision (*n* = 3/7).

*I can't even remember saying no* (…) *so, the only reason is really that I never get it done. It is that you must fill in something and then you have to send it back again and something like that, I don't get it done. (…) it should be happening on such a website, so you just push, make the questionnaire in there and press send, and then it is sent* (P3-DK, mother).

Most parents stated that postal questionnaires were a demotivating factor to participate (DK;IT;PT). They suggested a more flexible procedure: face-to-face, by phone or electronic form, and to assure that those questionnaires were short in length and straightforward (*n* = 5/7 DK; *n* = 2/3 IT; *n* = 11/12 PT). More opportunity for open-ended, “more personal” feedback, which was “more favourable to clarify doubts” was also advised.

All teams resorted to “reminders” to improve participation within follow-ups (DK;IT;PT). Parents who responded to all waves expressed that reminders and phone contact as commonly took place in the cohorts were facilitators for participation, while those that failed to participate to previous study waves manifested that researchers should have insisted in obtaining response. No parent mentioned that contacts or invitations to participate had ever been too insistent or impolite (DK;IT;PT).

*The people who called me were always of extraordinary kindness and gentleness and this is very important. Not too boring nor with constant phone calls, and I think that’s important, too.* (P5-PT, mother).

All parents who have participated in the 5-yrs neurodevelopmental assessment (PT;IT) expressed their appreciation for the chosen assessment method, for the opportunity of a face-to-face interaction with the researchers, the flexibility offered on timing and location, and the return of results. Parents in the Italian context, added that this was the follow-up which better met their expectations and should be replicated more often.

## Discussion

This study explored situational elements involved in the phenomena of participation and attrition in three European birth cohort studies of children born VPT and/or VLBW, while addressing the interplay of the points of view and lived experience of individuals in both standpoints of the research process. The process of retention begins at recruitment and the relationship between research teams and participants must be understood as a whole that needs to be dynamically sustained over many years. Where participants are enrolled in birth cohorts, parents are providing consent on behalf of their children. Study designs usually imply parents’ compliance in filling periodic questionnaires, participating in interviews and periodic child evaluations, at given intervals over a long-time span. It implies the sustained commitment by parents to participate in the study with their child. Though we consider that a greater involvement of children has both a rights-based dimension and potential benefit to research [21], it was their parents’ perspective that were determinant to understand participation during this life cycle of the cohorts. Our findings were dominated by female gender’s point of view and that is not a bias of our purposive sample. As reported by cohort management teams, it reflects the weight of the female gender’s involvement in these child cohort studies. This overrepresentation suggests that females’ perspective and lived experiences on participation are an influential factor and should be at the core of strategic management decisions on these child cohorts [22]. Moreover, in long-term cohort studies, participation is expected to continue beyond childhood. As also revealed by our study, efforts should be made to implement regular bond-building strategies overtime, without overlooking those directed to children in order to promote their long-term retention.

Altruism/solidarity along with gratitude/sense of duty to reciprocate were the concurrent main underlying motivating factors expressed by parents for enrolling and taking part in these studies. Lack of bonding and of identification with the cohort along with insufficient information on the study and its findings emerged as main motivational deterrents in all contexts. In other words, it discloses the perceived failure of the research stance to meet participants’ expectation of reciprocity/return. These same main motivating factors have been stated by multiple participants in various kinds of longitudinal studies and across social settings as one of the main reasons for participation [9]. Authors of these studies have attempted to interpret what seems to be a paradoxical concurrence of this motivation with the expectation of reciprocity by using concepts, such as ‘conditional altruism’, ‘weak altruism’ or ‘perhaps less truthful’, «in order to be more socially acceptable» [23]. Consent to enrol in these cohorts did not presuppose the use of direct incentives of any kind. It is therefore reasonable to interpret it ‘as truthful’ being indeed an act of gift-giving; thus, the generous transfer of socially valued objects without any guarantee of reciprocation. Moreover, the concept of altruism in health-related research was sometimes contested because it was framed by the belief that human altruism is a sole or overriding motivator dissociated from any kind of self-interest, self-protection or expectation. However, there is no contradiction to be identified in the association of altruism with the expectation to give in return. Reciprocity in gift exchange is framed as the expression of the social bond that contributes to the creation and balanced maintenance of relationships in society [24]. Our results show that, though there was no legal or contractual guarantee of reciprocation, a perceived unbalanced research relationship between those who altruistically give and those who accept led to adverse effects, namely: lack of bonding and increased careless- or non-response.

Our findings also revealed that the phenomena of participation and attrition was inextricably linked to parents’ lived experiences on having a VPT birth and on the support provided by the healthcare system. This perceived entanglement acted both as facilitator for enrolment and as deterrent for later study waves. As voiced by parents, their situational vulnerability during the enrolment of their newborn influenced positively their decision-making as it generated an ambivalent potentiality. Their lived experience at the time favoured a condition of openness, of receptivity to both ‘being affected’ and not even willing to question or retain detailed information on the study, and ‘affecting in turn’ by finding comfort in using their distressing experience for the benefit of others [25]. As their capacity for a generous transfer at such time was nonetheless limited, the strategy of abstracting data from medical records without requiring other parental response was a further sensitive facilitator to baseline participation.

As follow-ups proceed, however, continuous lived experiences of distressing parenthood when added to situations of vulnerability and of frustration with the healthcare system for not responding adequately to their needs may become a closing factor in the weighing process for the decision of non-response to yet another solicitation. The recurrence of feelings of vulnerability and being overwhelmed were common to all parents. Also, the complaint about the lack of healthcare provision and adequate psychological support for mothers was especially pointed, in the first 2 years, in all three countries. These included accounts of parents describing healthy children, stable relationships, good extended family support and comfortable economic situations. These findings suggest that these aspects of prematurity, mother psychological distress and general parental stress and coping, either have not yet received enough adequate attention from researchers, or findings are not adequately translated into healthcare policies and practices in Europe [26]. Besides adequate information sharing on the study to minimize misperceptions of clinical and research entanglement, as from the moment of recruitment, cohorts would benefit from promoting opportunities for a more participatory research process. As voiced by parents in all contexts, resorting to “more personal” methods of interaction, which enable clarification of doubts and discussion on the subjects of their affliction, further enhances feelings of “familiarity” and “closeness”, potentiating enduring engagement.

In all settings, the implementation of particular strategic procedures and analysis of parents’ narratives confirmed the correctness of inquired professionals’ perceptions regarding desirable traits of strategies to contact and interact with participants. In line with recent findings by Teague et al. [8], multiple and flexible, tailored strategies, particularly offering alternative methods of contact and data collection, favours participation, whereas financial incentives were not at all mentioned by any parent in the three European cohorts. Differing, our findings also add regularity of contact/interaction between researchers and participants as well as the use of reminders as major facilitators as found in Booker, Harding and Benzeval (2011). The key challenge seems to be calibrating the cost-effectiveness of reminder strategies appropriately against the benefits, to optimise response. Nevertheless, some studies suggest that participants’ response increased by sending at least one reminder to those who had not yet replied. Others suggest that pre-called participants are less likely to require a reminder or require fewer reminders, which was mirrored in our findings [3].

The phenomenological approach allowed to find strategic procedures that may have been influential factors towards participation and attrition and that would not have been identified by usual methods. The most common method to collect data - questionnaires sent and returned by post, was singled out by all parents as a demotivating factor to participate. They prefer flexible and replaceable methods. Parents advised to assure that questionnaires were short and straightforward with more opportunity for open-ended feedback, in line with other studies [20].

Our findings also revealed important situational challenges to the cohorts which affect the potential and range of possibilities for implementation strategies. All three settings share the impact of influx and mobility of populations within the last decade, related with European Union (EU) geopolitical and economic contexts, whereas Italy stands out for the much higher increase of foreign population density. Though all professionals shared long concerns that minorities and vulnerable groups are at higher risk of loss to follow-up in cohorts [27], the contextual environment severely restricts the implementation of strategies to address it. Prevailing model for research funding in Europe further restricts the possibilities to sustain desired regular contact in-between study waves, in organizing initiatives to increase a more participatory relationship, tailoring barrier-reduction strategies to vulnerable participants or to ensure constancy in research staff to promote identification with the study. Nevertheless, we were also able to find that opting for multipurpose strategies may help in calibrating cost-effectiveness of required procedures to overcome those challenges. The strategy of the PT cohort of sending a birthday postcard to children every year is exemplary in this regard.

Cohort studies exist in contextual material, environmental, social and political conditions and those also change over time. The use of large-scale, long-term cohorts, as proposed by RECAP Preterm project, has the potential to capture the value and differential effect of policy and program interventions that operate within and outside of the health sector to understand health quality and health equity. Routine monitoring systems are needed to «enable generation and sharing of new evidence on the ways in which social determinants influence population health and health equity and on the effectiveness of measures to reduce health inequities through action on social determinants» [28]. Our study findings suggest that it would be also necessary to capture the value and effect of policy and program interventions on the making of research in itself.

### Limitations

Multi-situated research using multimodal data collection entails increased effort, resources and time while increasing the complexity of analysis. This study limited its in-depth examination to three cohorts. Further similar primary research in more and diverse European VPT/VLBW existing cohort studies will be needed to expand our understanding.

## Conclusion

The multi-situated approach used, addressing the interplay of the lived experience of individuals in both research standpoints, was of most value to better understand variability and cost-effectiveness of different implemented approaches. Both cross-contextual and context-specific situational elements that have been influential factors towards participation and attrition in these cohorts were identified. European cohorts of children born VPT/VLBW may benefit from exploring these findings to develop novel and/or more ‘in context’ strategies to improve participants’ retention.

## Supplementary Information

**Additional file 1.**

## Data Availability

Multi-site datasets generated for this study cannot be shared for legal, ethical and privacy restrictions. In accordance with multi-site ethical clearances and signed informed consent provided by participants which guarantees their anonymity and confidentiality, generated data for this study may only be accessed and handled within RECAP Preterm-WP6 work group research team and under the framework of internal governance of the Horizon 2020 project RECAP Preterm funded by the European Union under grant agreement N° 733280.

## Abbreviations

DK: Denmark
EPICE: Effective Perinatal Intensive Care in Europe
EU: European Union
GA: Gestational age
Ps: Parents of children participating in the cohorts
PRFs: Healthcare and research professionals involved with the cohorts
IT: Italy
NICUs: Neonatal Intensive Care Units
PT: Portugal
RECAP Preterm: Research on European Children and Adults born Preterm project
SHIPS: Screening to Improve Health in very Preterm Infants in Europe
VPT/VLBW: Very preterm and/or very low birth weight
