# Supplementary material for: Understanding participation in European cohort studies of preterm children: the views of parents, healthcare professionals and researchers
Source: BMC Med Res Methodol. 2021 Jan 12;21:19. doi: 10.1186/s12874-020-01206-5 (PMC7802270; doi:10.1186/s12874-020-01206-5)
Supplement: Supplementary file 1 — Additional file 1. [file 12874_2020_1206_MOESM1_ESM.pdf]

RECAP preterm

Qualitative Study for Improving data collection, follow-up, and participant involvement

INTERVIEWING GUIDE - LIST OF COMMON KEY ISSUES TO BE ADDRESSED IN ALL STUDY SITES

Focus Group discussion or Individual semi-driven interviewing

Focused Questions:

- What are the motivating factors influencing participation?
- What are the demotivating factors influencing participation?
- How do the ways in which cohort studies' participants conceptualize and experience the study participation processes shape their participation?
- How does the lived experience ("lifeworld context", circumstances) and expectations of study participants and parents (caregivers) interplays with the responses obtained by the professionals in follow-up enquiries in different European cohort studies?

a) Interviewing with health-care professionals, cohort staff, and/or other relevant key-actors involved with VPT/VLBW cohort studies:

To be conducted either in the native-speakers' language or in English (in case the participant/all FG participants are proficient English speakers and the local researchers find it appropriate).

Key-issues to approach

FG discussions will be driven by the key-issues under study while including some in-context subtopics of discussion elected by partners as relevant to the specificities of their participants.

Open-ended questions on (6-8) key-issues to approach should be introduced if not spontaneously addressed by the participants. Suggestions:

1. Obstacles/problems faced in your specific context of involvement with the cohort study,

such as legal, professional, logistic or economical, pre-established procedures, *habitus*.

2. Most encouraging aspects of the procedures/activities related with your cohort involvement.
3. Most discouraging aspects of the procedures/activities related with your cohort involvement.
4. Do you feel empowered enough to make suggestions for the cohort management/study? Do you feel that you have appropriate feedback from the researchers? (Asking to reflect on personal experiences, not in a way for possible 'yes' or 'no' answers)
5. Do you feel that you have appropriate feedback on study findings? Are these results being integrated in you practices? (Asking to reflect on personal experiences, not in a way for possible 'yes' or 'no' answers)
6. Suggestions to improve participant enrolment and commitment continuity in long-term cohort studies in your particular context (professional, social, regional, national). What measures would you implement in an ideal scenario?

b) Interviewing with parents of children born VPT/VLBW participating in a cohort:

To be conducted in the native-speakers' language (in case of FG comprising foreign, immigrant, or different spoken languages' participants, the local researcher should choose the most inclusive language).

Key-issues to approach

Open-ended questions on (6-8) key-issues to approach should be introduced if not spontaneously addressed by the participants. Suggestions:

1. Personal experience (or known from others) of cohort study practices and procedures that facilitate/promote participants willingness to respond to follow-up activities. What procedures and methods of enquiry do you find most encouraging/discouraging?
2. Any personal experience (or known from others) of answering follow-up questionnaires in a rush, with answers not necessarily accurate, like "I am not sure, so I will answer this…, it will not make a difference"; or answers that you feel will look better in the questionnaire just to save time in completing it?
3. Obstacles/problems faced in your specific context of involvement with the cohort study, such as professional, economical, travel distances, social and family support /single parenthood, language, migration status, illness, disabilities.
4. Do you feel empowered enough to make suggestions for the cohort management/study?

Do you feel that you have appropriate feedback from the researchers? (Asking to reflect on personal experiences, not in a way for possible ‘yes’ or ‘no’ answers)

5. In your opinion, being a part of this cohort study has, has had, or may have any benefit or any disadvantage to you, to your loved ones or to the society in general? (What would make your opinion change?) / Do you feel that being part of a cohort has had any positive impact in your lives? Do you expect some positive impact in their future?
6. Are you involved in social support or social activist groups? Do you feel that you have appropriate feedback on research findings? Are these results being integrated in your lives? (Asking to reflect on personal experiences, not in a way for possible ‘yes’ or ‘no’ answers)
7. Any personal experiences (or known from others), life circumstances or difficulties faced by parents of children born preterm that you feel are never asked/discussed and yet would be important to be known by researchers in order to find more adequate strategies to meet your expectations?
8. Suggestions to improve participants’ commitment continuity in the cohort study. What measures would you implement in an ideal scenario?
